# Computational Prediction of Protein Intrinsically Disordered Region Related Interactions and Functions

**DOI:** 10.3390/genes14020432

**Published:** 2023-02-08

**Authors:** Bingqing Han, Chongjiao Ren, Wenda Wang, Jiashan Li, Xinqi Gong

**Affiliations:** 1 Mathematical Intelligence Application Lab, Institute for Mathematical Sciences, Renmin University of China, Beijing 100872, China; 2 Beijing Academy of Intelligence, Beijing 100083, China

**Keywords:** intrinsically disordered proteins and regions, interactions, functions, computational prediction

## Abstract

Intrinsically Disordered Proteins (IDPs) and Regions (IDRs) exist widely. Although without well-defined structures, they participate in many important biological processes. In addition, they are also widely related to human diseases and have become potential targets in drug discovery. However, there is a big gap between the experimental annotations related to IDPs/IDRs and their actual number. In recent decades, the computational methods related to IDPs/IDRs have been developed vigorously, including predicting IDPs/IDRs, the binding modes of IDPs/IDRs, the binding sites of IDPs/IDRs, and the molecular functions of IDPs/IDRs according to different tasks. In view of the correlation between these predictors, we have reviewed these prediction methods uniformly for the first time, summarized their computational methods and predictive performance, and discussed some problems and perspectives.

## 1. Introduction

Intrinsically disordered proteins (IDPs) refer to proteins that cannot be stably folded into a unique three-dimensional structure under physiological conditions [1,2,3,4], and they appear in the form of a conformational ensemble in solution [5]. IDPs/IDRs are widespread, and their proportion increases with the complexity of organisms [3,6], especially in eukaryotes [7,8]. In the eukaryotic proteome, more than 40% of proteins are intrinsically disordered or contain more than 30 amino acids of intrinsically disordered regions (IDRs) [1]. The research [9,10] show that, in the PDB dataset, the proportions of proteins/chains containing IDRs to total numbers of proteins/chains are about 50% and 55%, respectively (as shown in Table 1). Most of these IDRs are short disordered regions (SDRs), accounting for about 90% of all IDRs. Although there is no well-defined three-dimensional structure, IDPs/IDRs have normal biological functions and are widely involved in transcription, regulation, translation, cell signal transduction, protein aggregation, protein phosphorylation, small molecule storage, and other important biological processes [4]. Furthermore, disordered segments often act as flexible linkers between folded domains in multidomain proteins [11]. They also participate in molecular recognition, molecular assembly, and protein modification through protein–protein, protein–nucleic acid, and protein–ligand interactions [12]. The discovery of disordered proteins is a challenge to the traditional protein “sequence–structure–function” paradigm. IDPs/IDRs usually have the primary structural characteristics of low sequence complexity [13], high repeatability, low content of hydrophobic amino acid residues, and high content of charged and polar amino acid residues [4]. Their dynamic conformation allows proteins to interact with multiple targets [14], and they have the biological advantages of easy binding, spatial superiority, and high coordination. In addition, IDPs/IDRs are widely related to human diseases, such as cancer, genetic diseases, cardiovascular diseases, amyloidosis, neurodegenerative diseases, synucleinopathies, and Alzheimer’s disease [15,16,17], which makes them potential targets for drug discovery [18,19,20]. Undoubtedly, we cannot ignore these relatively large and functionally important IDPs/IDRs in the proteome [21]. Understanding these proteins will help us to further understand the importance of disordered proteins and their molecular mechanisms involved in physiological and pathological processes and help us to understand some human diseases and develop drugs.

However, IDPs/IDRs and their interactions and functions in those currently annotated by experiments are few, and there is still a big gap with their actual number. For example, some studies have shown that only 0.1% of the 147 million proteins sequenced have experimental annotations of intrinsic disorder [22]. This is mainly because the experimental identification is time-consuming and it is difficult to carry out in a high-throughput way [15]. Based on the importance of IDPs/IDRs and the huge gap between the experimental annotations and the actual number, many computational methods related to IDPs/IDRs have been proposed. According to the different tasks, they can be divided into IDP/IDR predictors, predictors of binding modes of IDPs/IDRs, predictors of binding sites (regions) of IDPs/IDRs, and predictors of functions of IDPs/IDRs. Although the specific tasks to be implemented are different, these predictors have some associations. For example, the predictors of the binding sites of IDPs/IDRs often use the output results of IDP/IDR predictors as input information, and these predictors designed for different tasks all focus on the disordered regions and use similar feature sets and models. Some predictors can even implement multiple tasks at the same time. For example, flDPnn [23] couples disorder prediction and function prediction. These predictors can realize high-throughput prediction and make up for the shortcomings of experimental identification. From the perspective of computational method types, they include scoring function-based method, machine learning-based method, meta predictor, and template-based method.

In view of the correlation between these predictors, we first reviewed these prediction methods designed for different targets, including IDP/IDR prediction, the prediction of binding modes of disorder, the prediction of disordered binding regions, and the prediction of the functions of a disorder. We have summarized the computational methods used by these predictors and their predictive performance and discussed the problems to be solved and aspects to be improved in different tasks.

## 2. Predictions of Intrinsic Disorder

IDPs/IDRs exist widely. The data show that there are a large number of proteins with disorder in the PDB. The content of proteins/chains with disorder in the PDB database has been studied in the existing literature (as shown in Table 1). The dataset of Monzon et al. [9] and PDBS25 [10,24] show that in the PDB database, the proteins/chains with disorder accounted for 51.08% and 56.91% of the total proteins/chains, respectively. Among them, disordered residues accounted for 5.07% of the total residues. However, the influence of the number of chains of the proteins on disorder has not been explored in the existing literature, and we have made simple statistics on this. We selected the non-homologous nine-body proteins and seven-body proteins in PDB, respectively, which are resolved by using the X-ray diffraction method, with high refinement resolution, and each chain length is more than 100. We produced statistics on their disorder. As shown in Table 1, for seven-body proteins and nine-body proteins, the disordered residues accounted for 5.22% and 5.98%, respectively. This simple statistic shows that disorder may have some relationship with chain numbers of proteins, which requires further research and verification in combination with disorder prediction.

Many experimental techniques have been used to identify IDPs/IDRs, such as nuclear magnetic resonance (NMR) [25], X-ray crystallography [26], circular dichroism (CD) spectroscopy [27], small-angle X-ray scattering (SAXS), and single-molecule fluorescence resonance energy transfer (smFRET) [15], of which the first two are the most important [15].

At present, some special databases have provided annotations of IDPs/IDRs, including MobiDB [28], DisProt [21], IDEAL [29], etc. DisProt [21] is one of the most commonly used databases of disordered proteins, which mainly includes manually extracted IDPs/IDRs verified by NMR and X-ray crystal diffraction experiments. MobiDB [28] includes both disorder prediction and annotation. In addition, experimentally verified or predicted IDR annotations are also included in some core databases, such as InterPro [30], UniProt [31], PDB (missing electron identities in X-ray analytical structure or the highly structurally varied regions in the nuclear magnetic resonance model) [32,33], and PDBe [34]. These data have been used to develop some computational methods for identifying IDPs/IDRs, which is helpful for experimental verification and thus promotes the discovery and expansion of data [34].

However, the experimental method is long-period and expensive, which is difficult to conduct in a high-throughput way [15], and there are also technical problems, and the gap between experimentally annotated proteins and non-annotated proteins is growing rapidly [35]. Therefore, it is necessary to develop the computational method for IDP/IDR prediction.

The first predictor was proposed by Williams 1979 [36]. In the following decades, many computational methods have been proposed for IDP/IDR prediction. These methods can be divided into two categories: one is at the protein level, which is used to predict whether an unknown protein or protein region contains disordered regions [37], and the other is at the amino acid residue level, given an unknown protein sequence to predict the probability to be disordered/ordered of the residues at each position of the sequence. At present, most studies are mainly conducted for the second category, and the prediction results can be further used to determine whether the whole protein is IDP or whether the protein contains disordered regions.

Some reviews have summarized and analyzed the development of IDP/IDR predictors from different perspectives [15,22,38,39,40,41,42,43,44,45,46]. For example, He et al. [41] introduced the prediction methods before 2009, summarized these different methods, analyzed their strengths and shortcomings, and discussed the difficulties and future development direction of disorder prediction. Meng et al. [46] not only reviewed some prediction methods from 2003 to 2015 but also summarized the predictors of the molecular functions of disorder. Liu et al. [15] reviewed the prediction methods from 2001 to 2017, classified and summarized their advantages and disadvantages, and made a comprehensive comparison of the predictive performance of the predictors based on CASP10 and the widely used benchmark datasets. Considering that the previous evaluation methods are based on the dataset level, Katuwawala et al. [22] evaluated the performance of 13 popular disorder predictors at the protein level for the first time. Katuwawala et al. [45] also released a new benchmark dataset, focusing on the prediction of protein and nuclear acid-binding proteins, and studied the impact of sequence similarity and experimental verification annotation on prediction quality. In recent years, the research on disorder predictors has been further developed.

### 2.1. Methods

According to the literature [15,46], the computational methods of IDP/IDR prediction can be divided into four categories, including the scoring function-based method, the machine learning-based method, the meta predictor, and the template-based method. According to the above classifications, we introduced the predictors belonging to different categories below. We have summarized some recent (after 2017) predictors in the Table 2, including their method categories, classifiers, features, whether to use evolutionary information, and applicable IDR types. As shown in Table 2, most of these methods are based on machine learning, especially on deep learning, which benefits from the increase in annotations and the development of deep learning technology. All of the websites of these methods have been last accessed on 29 December 2022. It can be seen from the table that most methods can predict all types of IDRs (without distinguishing in advance), while some need to select predicting LDRs or SDRs in advance, such as IUPred3 [47], and some are more suitable for predicting one type of IDRs such as DisoMine [48].

#### 2.1.1. Scoring Function-Based Methods

This method is based on the physicochemical characteristics of amino acid residues to form a function or formula for calculating the disorder tendency [46]. These physicochemical properties include specific amino acid composition, electrostatic charge, hydrophobicity, residue–residue contact, predicted secondary structure tendency and solvent accessibility, etc. Such methods include GlobPlot [49], NORSp [50], IURed [51,52], FoldIndex [53], FoldUnfold [54,55] etc., most of which were concentrated before 2007.

Some methods take into account the lack of secondary structure in IDRs and use the structure information to calculate the disorder tendency. For example, GlobPlot [49] calculates the parameter P=RC−SS describing disorder tendency through RC and SS, where *P*, *RC*, and *SS* are the propensity for a given amino acid to be in disordered state, ‘random coil’ and ‘secondary structure’. The basic algorithm behind GlobPlot is a sum function Ω of P. NORSP [50] calculates the secondary structure content of the sequence window by combining the predicted secondary structure, membrane helices, and coiled-coil information. The region whose structure content is lower than the given threshold is identified as disordered.

There are some methods used to construct algorithms from the perspective that IDRs have different amino acid compositions compared to ordered residues. For example, IUPred [51,52] is based on the assumption that the disordered protein has a special amino acid composition, which prevents it from forming a stable conformation. This method calculates the pair interaction energy from known coordinates, where the energy of each residue is estimated based on the amino acid type, amino acid composition and its sequence neighborhood. Thus, the disordered residues can be predicted as corresponding regions with unfavorable estimated energy. IUPred2 [56] implements several minor bug fixes based on IURed. IUPred3 [47] improves predictive performance through multiple new smoothing functions and a new dataset consisting of experimentally verified ordered/disordered regions. The FoldIndex method [53] is based on the mean residue hydrophobicity 〈H〉 and net charge of the sequence |〈R〉|. The index FoldIndex is calculated by a linear formula of 〈H〉 and |〈R〉|, and its negative value corresponds to IDPs.

The principle of scoring function-based methods is clear, and their formula is concise. The prediction results of these methods are easy to explain and can build trust in researchers’ decision-making, but there are also shortcomings. Most of these methods are not complex and predict disorder by a simple linear formula or a given threshold. In some cases, they are difficult to fit the actual situation, resulting in predictive performance that is generally not as good as machine learning-based methods and meta-predictors.

#### 2.1.2. Machine Learning-Based Methods

In recent years, with the vigorous development of machine learning algorithms, many IDP/IDR predictors based on machine learning have been proposed. The machine learning-based methods use sequence-related features as inputs to predict the tendency of each residue in the sequence to be disordered. Furthermore, machine learning-based methods can be divided into classification models and sequence labeling models [15].

1. Classification models

For classification models, information relating to the target residues and their neighbor residues (the subsequences covered by the sliding window with the target position as the center) is usually represented as a fixed-length feature vector, which is input into some traditional machine learning models such as support vector machine (SVM), Random Forest (RF), logical regression (LR), fully connected neural network, and other models, and the final model is obtained by training with a large number of positive and negative samples; thus, the tendency of each residue to be disordered can be predicted. Input features mainly include (i) sequence information such as amino acid composition (AAC); (ii) evolutionary information such as Position-Specific Substitution Matrix (PSSM), hidden Markov model (HMM) profile; (iii) structural information, such as the predicted secondary structure [57,58].

The research of Romero et al. [59] shows that IDRs with different lengths have different features. That is, the short and long disordered regions of proteins have different preferences for different amino acid residues. Predictors trained on a specific type of IDRs cannot predict other types of IDRs well. Therefore, there are some predictors specific to certain types of IDRs (such as LDRs, IDRs with length ≥ 30 residues; and SDRs, IDRs with length < 30 residues). For example, POODLE-L [60], DRAai-L [57], PONDR VL3 [61], PONDR VL3H [61], PONDR VL3P [61], PONDR VL3E [61], SLIDER [8], and PONDR VL-XT [13,62] are specially designed to predict LDRs, POODLE-S [63], and DRAai-S [57] are specially designed to predict SDRs.

PONDR VL-XT [13,62] trains three different neural networks for the N terminal, C terminal, and internal region based on specific datasets, and the final result is the prediction result of each predictor in its corresponding region. Its input features include flexibility, hydropathy, coordination number, net charge, and specific amino acid composition. PONDR VL3 [61] combines local sequence complexity K2 entropy, amino acid frequency and flexibility as input features and uses an ensemble of neural networks as prediction models, which is better than the VL2 version that uses a simpler regression-based model [64]. Considering that VL3 uses a rather small dataset, VL3H [61] enhances the training set by adding homologous proteins of disordered sequences. Other settings include the features and models that are the same as VL3. VL3P [61] adds PSSM to improve the predictive performance. VL3E [61] combines VL3H and VL3P and uses ensemble neural networks to make final predictions through majority voting. Considering the lack of methods to directly predict whether LDRs are included at the protein level, SLIDER [8] uses a set of empirically selected features, including the physicochemical properties of amino acids, sequence complexity, and amino acid composition as inputs, and ridge regression model as the prediction model, which can accurately and quickly predict proteins with LDRs.

For the methods designed for SDRs, POODLE-S [63] defines seven regions from the N-terminal based on the fact that amino acid composition has different tendencies in the N-terminal, C-terminal and internal regions; each of the regions correspond to specific physicochemical features as input and an SVM as a prediction model. DRAai-S [57] selected features related to disorder from the amino acid index (AA index) database AAindex and used RF as the classifier.

Due to the poor performance of methods specially designed for LDRs/SDRs when predicting other types of IDRs, there are some general IDP/IDR predictors that do not specifically distinguish between LDRs and SDRs. This general method can be further divided into two categories, one is to combine the predictors specially designed for LDRs or SDRs for final prediction, and the other is to train all types of IDRs at the same time during training so that the predictors are universal [15]. The first type of methods includes PONDR VSL1 [65], PONDR VSL2 [66], SPINE-D [67], MFDp [68], etc. For example, PONDR VSL1 [65] and VSL2 [66] try to merge the LDR predictors and SDR predictors for the first time. VSL1 and VSL2 are two-level models; that is, two special predictors are established and optimized, respectively, for LDRs and SDRs at the first level, and the two special predictors are combined by the second-level meta predictors. SPINE-D [67] includes two hidden layers and an additional layer to smooth the prediction. First, a three-state prediction is performed at the residue level: ordered residues, residues belonging to SDRs, and residues belonging to LDRs. Then, by adding the probabilities belonging to SDRs and LDRs, the prediction is simplified to a two-state prediction. The input features of SPINE-D include residue-level and window-level information, wherein residue-level information includes physical parameters, PSSM, predicted secondary structure, predicted solvent accessibility, and predicted torsional angle fluctuation; window-level information includes amino acid composition, local composition complexity and predicted secondary structure content.

As for the second type, for example, svmPRAT [69] encodes the characteristics of the target residues and their neighbor residues as feature vectors with fixed lengths and adopts a flexible sequence window coding scheme, that is, weighting according to the distance between the adjacent residues and central residues. SvmPRAT combines PSSMs, BLOSUM62, and the predicted secondary structure as the feature set and uses SVM with the second-order exponential (soe) as the kernel. The results show that this flexible coding has some value in the disorder prediction problem. DisPredict [12] uses input features, including the amino acid type, physicochemical properties, PSSM, predicted secondary structure, predicted solvent accessibility, predicted backbone dihedral torsion angles fluctuations, and new Monogram and Bigram features calculated from PSSM, which uses single SVM with RBF kernel as the classifier. The fIDPnn [23] method uses some tools to generate the structure and function information to encode the sequence features, integrates the profiles at the residue, window, and protein levels, and then inputs the features into the deep learning model (DNN) for prediction. The innovation of fIDPnn lies in the introduction of the prediction of the functions of disorder and feature coding at the protein level as input information, which contributes greatly to the predictive performance. FlDPlr [23] uses the same framework as fIDPnn, in which the deep neural network is replaced by an LR model, it still shows good predictive performance, which indicates that good predictive performance mainly depends on the input profile and the features.

Although these methods have achieved great predictive performance, they only consider the dependence between the local residues, lack characterization of global sequence information, and do not utilize the global correlation between the residues.

2. Sequence labeling models

The input of the sequence labeling models is an unlabeled sequence (not only the information of the target residues and their neighbor residues), and the output is a sequence labeled with a disorder tendency; that is, each residue in the sequence is predicted. Such methods often use some deep learning technologies, such as Recurrent Neural Networks (RNNs), Convolutional Neural Networks (CNNs), Conditional Random Field (CRF), Long Short-Term Memory (LSTM), etc. AUCpreD [58], DISpro [70], OnD-CRF [71], DeepCNF-D [72], SPOT-Disorder [35], SPOT-Disorder-Single [73], SPOT-Disorder2 [74], DisoMine [48], etc., belong to this category of methods. For example, AUCpreD [58] trains Deep Convolutional Neural Fields (DeepCNF) by maximizing AUC, which is conducive to dealing with the problem of an unbalanced distribution in the disordered and ordered residues. Because the sequence profile generation takes time, AUCpreD has two prediction modes depending on whether the sequence profile is used. SPOT-Disorder [35] first applied LSTM to disorder prediction, using evolutionary information, predicted structural properties and physicochemical properties as input. The first layer of the network was a recurrent feed-forward layer followed by two LSTMs. Although SPOT-Disorder did not carry out specific training, it was able to process residues in disordered regions with different lengths and had the ability to predict functional sites in disordered regions. This success could be attributed to the ability of LSTM to recognize non-local interactions. Considering that the existing methods are divided into those that only rely on the single sequence and those that rely on evolutionary sequence profiles generated by multiple sequence alignment (MSA), the latter is more accurate but time-consuming. SPOT-Disorder-Single [73] focuses on improving the accuracy of prediction without relying on evolutionary information, using a binary one-hot matrix as the input and an ensemble of ResNets with LSTM networks as the prediction model. SPOT-Disorder-Single is more accurate than SPOT-Disorder in predicting proteins with few homologous sequences. The input features of SPOT-Disorder2 are similar to SPOT-Disorder, whose neural network topology is composed of various models combining Squeeze-and-Excitation networks (IncReSeNet), LSTM and Full Connect (FC). Compared with SPOT-Disorder, SPOT-Disorder2 [74] provides substantial and consistent improvements, this is mainly due to the improvement of the neural network model. Different from other predictors whose inputs are pre-determined features, rawMSA [75] takes the whole MSA as the text input of the neural network, which is mapped from amino acid letters to floating point vectors by an embedding layer. Considering that previous predictors did not use sequence-dependent emergent properties, DisoMine [48] uses GRU to predict LDRs based on protein dynamics, predicted secondary structure and predicted early folding.

Different from the classification models, the sequence labeling models can effectively use the global sequence information rather than only the local sequence information.

The methods based on machine learning use more complex models and show better predictive performance, but their corresponding interpretability is insufficient. In addition, some of these methods use evolutionary profiles, such as PSSM and HMM profiles, whose generation is time-consuming and increases the running time of the model [58].

#### 2.1.3. Template-Based Methods

These methods search for homologous proteins with known structural information (i.e., templates) and predict the disorder of unknown proteins [15,46]. These methods include PrDOS [76], DISOclust3 [77], GSmetaDiscorder3D [78], etc. PrDOS [76] consists of two predictors, one based on local amino acid sequence information and the other based on template protein. The latter is based on the conservative assumption of intrinsic disorder in the protein family. PSI-BLAST is used for sequence alignment, and the weighted average of the alignment results that meet the conditions is used to predict whether the residues are disordered. GSmetaDiscorder3D [78] considered different fold recognition methods and used genetic algorithms to optimize the weight of a single method based on alignment quality. DISOclust3 [77] uses a multi-template modeling method and improves performance through additional sequence–structure alignment methods.

Template-based methods are easy to explain, but in general, it may be difficult to find high-quality templates for prediction, and it is generally required to use in combination with other methods.

#### 2.1.4. Meta-Predictors

Considering that different predictors use different sequence features, prediction models, and training sets [79], which are complementary, these methods combine the outputs generated by different disorder predictors as inputs to produce the final prediction results. Compared with the single-component predictor, the meta-predictor can further improve the predictive performance. Combination methods include simple voting, linear combination, or a nonlinear combination, such as the machine learning model. For example, MD [80], MetaDisorder [78], disCoP [81], DisMeta [82], PONDR-FIT [79], Spritz [83], MFDp [68], ESpritz [84], metaPrDOS [85], MFDp2 [86], DISOPRED3 [87], MobiDB-lite [88], etc., are meta-predictors. For example, PONDR-FIT [79] is a combined predictor based on PONDR VLXT, PONDR VL3, PONDR VSL2, IUPred, FoldIndex, and TopIDP. MetaDisorder [78] considers the results of 13 disorder predictors, and the final prediction was weighted by the accuracy of these methods. For each target residue, PONDR-FIT [79] inputs the prediction results of six predictors of this residue and surrounding residues into the artificial neural network to obtain the final prediction. The disCoP [81] aggregates the outputs of seven single predictors through 11 sliding window-based features and inputs them to the binomial deviance loss-based regression model to achieve consensus prediction.

These methods can often produce better predictive performance due to the combination of different disorder predictors. The disadvantages of these kinds of methods are the large number of calculations required and the long running time.

### 2.2. Predictive Performance

Some performance measures are widely used for evaluating the predictive performance of predictors, including area under ROC curve (AUC), balanced accuracy (BAC or Acc), Matthews correlation coefficient (MCC), and F1-score (F1-s). AUC is defined as the area enclosed by the coordinate axis and the ROC curve, which returns a value between 0 and 1. Others are defined as follows:

$$BAC=\frac{1}{2}\left(\frac{TP}{TP+FN}+\frac{TN}{TN+FP}\right)\;MCC=\frac{TP\times TN-FP\times FN}{\sqrt{\left(TP+FP\right)\left(TP+FN\right)\left(TN+FP\right)\left(TN+FN\right)}}\;F1-s=2\cdot \frac{precision\times recall}{precision+recall}$$

where TP (true positive) is the number of correctly predicted disordered residues; TN (true negative) is the number of correctly predicted ordered residues; FP (false positive) is the number of ordered residues that are predicted as disordered; FN (false negative) is the number of disordered residues that are predicted as ordered; precision and recall are defined as follows:

$$precision=\frac{TP}{TP+FP}\;recall=\frac{TP}{TP+FN}$$

Critical Assessment of protein Structure Prediction (CASP) 5 first carried out a community-based assessment of disorder prediction in 2002, among which six groups predicted disorder [89]. As a part of the CASP experiment, the disorder prediction evaluation continued until CASP10 [90], in which 28 disorder predictors were evaluated. Critical Assessment of protein Intrinsic Disorder prediction (CAID) is a biannual blind test to determine the latest level of prediction of IDRs and residues involved in binding. The first CAID experiment evaluated 32 disorder predictors on 646 protein datasets from DisProt, among which the best method is to use deep learning technology, which is significantly better than the physicochemical methods [18].

According to different sources of negatives, CAID involves two datasets when evaluating disorder prediction: one is the DisProt dataset, where the negatives are all residues outside the annotation regions, and another is the DisProt-PDB dataset, where the negatives are restricted to the PDB observed residues. CAID also gives the calculation results of metrics at residue level (per-residue classification) and protein level (per-protein classification), respectively. The residue level is used to view the dataset as a whole, and the protein level is used to calculate the metrics of each protein and take the average value. We compared the performance of the predictors with different datasets and measures calculation methods. As shown in Table 3, the per-residue classification performance of the predictors is better than per-protein classification, whether in the DisProt dataset or the DisProt-PDB dataset. This is further verified by the use of the Wilcoxon signed-rank test: different evaluation methods show significant differences in MCC and BAC (*p*-value < 0.05) on both datasets, which means that per-residue and per-protein classification are different in MCC and BAC. This difference may be due to the fact that some proteins, such as DP01898, DP01324, etc., are difficult to predict, on which almost all predictors secure F_Max_ close to 0, thus reducing the average level of performance. The different performance of the predictors on different proteins suggests that some proteins may need to be paid extra attention to improve the adaptability of the predictors. The performance of the predictors on the DisProt-PDB dataset is generally better than that in the DisProt dataset, especially the per-residue classification performance on the two datasets has significant differences in BAC, MCC, and F1 (*p* values = 2.026 × 10^−7^, 6.328 × 10^−9^ and 1.2 × 10^−10^, respectively) verified by Wilcoxon signed-rank test. This may be due to incomplete annotation, which increases the number of false positives [18].

For the AUC, flDPnn secures the best AUC = 0.814 in the DisProt dataset, and the ten top-ranking methods all secure AUC ≥ 0.74. While on the DisProt-PDB dataset, SPOT-Disorder2 secures the best AUC = 0.92, and the ten top-ranking predictors all perform AUC ≥ 0.86. According to the AUC value and Table 3, some current disorder predictors have provided high-quality predictions. Using different performance measures, some methods, including SPOT-Disorder2, fIDPnn, RawMSA, and AUCpreD, continue to perform well and always rank in the top five [18]. These four predictors are all based on deep learning, and most (except for flDPnn) are sequence labeling models. Their good performance may be due to the deep learning technology, which can extract complex relationships. In addition, the sequence labeling models can fully utilize global sequence information to capture the interdependence between distant residues. Although it does belong to the sequence labeling model, flDPnn also extracts protein-level information, which contains global sequence information.

In addition to IDR prediction, the prediction of fully disordered proteins is a challenge that deserves separate attention. Table 3 also shows the performance of the predictors in identifying fully disordered proteins (at least 95% of residues are predicted or annotated as disordered) evaluated by CAID1. FlDPnn and RawMSA show advantages in identifying fully IDPs and secure F1-s = 0.598, 0.578, respectively, while the third-best predictor performs F1-s = 0.505.

From Table 3, we can see that although some methods can show relatively continuous good performance, none of them can always hold high-quality performances under different evaluation methods. Therefore, it is necessary to fully consider the use purpose and the characteristics of predictors in practical application. For example, RawMSA has obvious advantages in predicting full IDPs, and some methods (such as flDPnn) have a shorter running time.

### 2.3. Problems and Perspectives

At present, IDP/IDR predictors have reached a relatively mature state [18], but there are still some problems to be solved and areas to be improved.

In terms of data, the information provided by different experimental techniques to detect IDPs/IDRs is slightly different [15]. Because the structure state of a protein is a continuum, including ordered, collapsed disordered, and extended disordered, the definition of disorder is not accurate yet [81]. Due to these different experimental techniques and unclear definitions, there is a lack of a unified experimental standard to identify disorders [18]. It is clear that IDPs/IDRs identified by various experimental technologies are more reliable. At present, the number of IDPs/IDRs identified by these nonuniform experimental methods is limited [15]. At the same time, another problem with the data is the acquisition of negative samples (i.e., ordered residues) [18]. The quality of negative samples in the benchmark dataset needs to be improved, and higher-quality order annotations are needed. At the same time, the current benchmark dataset lacks a fully structured protein, and the prediction quality is sensitive to it [45]. This protein can also be included in the training set to reduce the false positive rate [45,81]. For the prediction of IDPs/IDRs, it is necessary to establish a more comprehensive and accurate benchmark dataset.

In terms of software upgrading, the results of CAID1 show that the execution time of different methods can differ by four orders of magnitude [18], indicating that there is also a large space for optimization in the running time.

With regard to prediction targets, existing methods cannot well identify fully disordered proteins [18] and proteins with a large amount of disorder, such as disordered protein-binding proteins [45]. Therefore, predictors can be specially trained to identify these proteins. The above analysis shows that the disorder predictors may need more detailed training to adapt to different types of IDPs/IDRs. On the other hand, most of the current methods only focus on two classifications: ordered and disordered. Only a few methods have considered the semi-disorder of proteins, such as SPINE-D, SPOT-Disorder, and SPOT-Disorder2. Some studies have proposed the hypothesis that protein may exist in one of three forms: ordered(fully-folded), collapsed disordered (molten globule-like), or extended disordered (random coil-like) [61,91]. In addition, another study [92] proposed the Protein Quarter model; that is, the protein conformation is divided into four specific conformations: ordered forms, molten globules, premolten globules, and random coils. There are differences in the conformations of IDPs/IDRs, but there are few predictors that focus on such detailed classification of IDPs/IDRs.

As for the quality evaluation of disorder prediction, the current disorder prediction does not include quality assessment (QA) scores. Quality assessment tools can show which disorder predictors are more reliable. Ideally, correctly predicted disordered residues correspond to higher QA scores, and incorrectly predicted residues have lower QA scores. The recently developed QUARTER [93] and its improved version QUARTERplus [94] can evaluate the quality of 10 different disorder predictors. This QAtool needs further development to help users make better use of various disorder predictors.

In terms of application expansion, the disorder predictors can be integrated into other tasks. For example, in protein tertiary structure prediction methods, there are few special treatments for disordered regions, such as labeling or removing possible disordered regions. Therefore, we can consider integrating disorder prediction into protein three-dimensional structure prediction [81]. Similarly, disorder predictors can be combined with PPI prediction, phosphorylation site prediction and other tasks.

## 3. Interaction Prediction of IDPs/IDRs

IDPs can interact with a wide range of molecules, such as proteins, nucleic acids, and lipids, and mediate many important biological processes, including signal transduction and regulation [95,96,97,98]. Hub proteins, defined as proteins that interact with a large number of proteins in the PPI network, have a higher tendency to be disordered than other proteins [64,99]. The structural flexibility of IDRs enables them to ideally adapt to the binding surface of their target domain [100] so that they can interact with many partners and fold into different conformations when combined with different partners [101]. This conformational plasticity provides IDPs/IDRs with more extensive special functional advantages than the functional model of ordered proteins, thus complementing the inherent functions of ordered proteins [101,102,103].

In most of these interactions, IDPs/IDRs adopt stable binding structures; that is, the disordered proteins in the free state adopt well-defined structures in the binding form, that is, coupled folding upon binding [104,105]. In recent years, some IDPs have been found to be able to form “fuzzy complexes”; that is, IDPs are still in a disordered state after binding ligands to form complexes. Most fuzzy complexes are formed by a disordered protein and a structural protein, while the more extreme case is that both are disordered proteins [106].

The interaction information of IDRs/IDRs is stored in various specific datasets. DisBind [107] stores the protein-binding sites of IDPs supported by experiments. FuzDB provides a wealth of examples of experimentally determined fuzzy interactions [108]. Eukaryotic Linear Motive (ELM) resource mainly involves IDP-ordered protein interactions [109], in which experimental verified short linear motifs are stored, including 317 motif types and 3934 separate motif instances [110]. DIBS [95] stores the complexes formed by IDPs and globular/ordered partner proteins and provides corresponding dissociation constants, linear motifs involved in binding, functional annotation, etc. As a sister database of DIBS, MFIB [109] stores IDP-only complexes and their structural and functional annotations.

### 3.1. Prediction of Binding Modes of Intrinsically Disordered Proteins

In previous research (before 2020) focusing on computational methods, most of them only considered the disorder-to-order in the binding modes but could not characterize the continuum of interaction modes [104].

In order to predict the binding modes, Miskei et al. [104] constructed three data sets, including 828 regions of disorder-to-order, disorder-to-disorder, and context-dependent transitions (both disorder and order forms exist in the complex), that is, DOR, DDR, and CDR, respectively. They developed the FuzPred method based on the local sequence biases (the local biases between the site R and its flanking region on the disorder, composition and hydrophilicity), predicting the binding modes without any information about the partner. FuzPred applied a binary logistic model to predict the probability of transition from disorder to order $p_{DO}\left(A_{i}\right)$ and disorder to disorder $p_{DD}\left(A_{i}\right)$ $\left(p_{DD}\left(A_{i}\right)=1-p_{DO}\left(A_{i}\right)\right)$ for a given residue $A_{i}$. $A_{i}$ with higher $p_{DO}\left(A_{i}\right)$ is more likely to belong to DOR, and those with lower values are more likely in DDR. When $p_{DO}\left(A_{i}\right)$ and $\left(1-p_{DO}\left(A_{i}\right)\right)$ are comparable, $A_{i}$ belongs to CDR. The FuzPred method provides a continuous scale to characterize the binding modes of disordered regions [104].

Furthermore, based on conformational entropy, Fuxreiter [111] proposed a classification scheme of IDR interactions using the FuzPred method. Specifically, the FuzPred method is used to calculate the transition probability distribution of disorder-to-order or disorder-to-disorder and its Shannon entropy, which defines the entropy of the binding mode ($S_{bind}$). The “entropy” of the binding mode tells us to what extent the binding mode is clearly defined (the fuzzy binding mode has a high entropy value, and the consistent binding mode has a low entropy value) and to what extent it will change with the cell conditions.

### 3.2. Prediction of Binding Sites (Regions) of IDPs/IDRs

IDRs are rich in binding sites, which can play many important biological functions, including signal transduction and regulation by interacting with different molecules. IDRs can interact extensively with proteins, DNA, RNA, lipids, and various small molecules [12]. However, only a few hundred interactions have been annotated in experiments, and this gap has promoted the development of computational methods for disordered binding sites [112,113]. Different partners of IDRs may lead to different characteristics of binding sites. Therefore, a number of predictors have been developed, targeting specific types of binding partners of disordered proteins.

At present, most methods focus on protein–protein interactions, although predictors aiming at disordered DNA, RNA, and lipid binding regions have also been developed. The development of these predictors will help to accelerate the PPI discovery process, such as MoRF prediction has been used for this purpose [101]. The features used by predictors for binding sites of disorder are similar to IDP/IDR prediction, including amino acid composition, sequence complexity, flexibility, physicochemical properties of amino acids, evolutionary information, structural information, etc. In addition, most predictors for binding sites also use disorder predictions as input features.

According to the different types of binding sites, predictions can be further divided into MoRF prediction, SLiM prediction, prediction of disordered protein-binding regions, LIP prediction, etc. The latest review [64,114] investigated about 20 computational predictors for disordered binding regions, most of which are targeted at MoRFs, and discussed the future development direction of these tools. Nowadays, the predictors of disordered binding regions have been further developed, and we investigated and analyzed them.

#### 3.2.1. Methods

1. Prediction of MoRFs

Molecular recognition features (MoRFs) are short binding regions located in long IDRs (most of which are 5–25 residues in length, up to 70 residues in length), undergo disorder-to-order transitions when binding with proteins and peptides, and stabilize by binding with partners [115,116,117,118,119]. Due to their flexible structures, MoRFs can accurately combine with their partners and play an important role in signal transduction and regulation [119]. The MoRF region can be divided into four types: α-MoRFs, folding into helices; β-MoRFs, folding into β chains; γ-MoRFs, folding into coils and complex-MoRFs, folding into regions with multiple secondary structures [64]. Compared with flanking IDRs, the amino acid composition and physicochemical properties of MoRFs show different tendencies, such as containing higher concentrations of large hydrophobic side chains.

In recent years, some MoRF predictors have been developed successively. The first method is α-MoRFpred [116,120], proposed in 2005 to predict α-MoRFs. First, α- MoRFpred uses heuristics to detect potential MoRF regions and then develops a neural network to identify real MoRFs in potential MoRFs based on features such as disorder predictions, secondary structure predictions, and amino acid index. Similar to disorder prediction, MoRF prediction methods can be divided into scoring function-based method, machine learning-based method and meta predictor.

For the scoring function-based methods, a representative example is Retro-MoRFs, which combines sequence alignment and disorder prediction for the first time to improve the reliability of identifying MoRFs. This takes into account the sequence similarity between the MoRFs to be predicted and the known MoRFs or their reversed sequences [100].

Machine learning-based methods can be further divided into traditional machine learning-based methods and deep learning-based methods. For the traditional machine learning-based methods, the models used include SVM, Naïve Bayes algorithm, and neural network, among which the most used model is SVM (As shown in Table 1, nine predictors used SVM). For example, MoRFpred [117], MFSPSSMpred [121], MoRF_CHiBi_ [122], DISOPRED3 [87], fMoRFpred [101], Predict-MoRFs [123], MoRFPred-plus [124], etc., are all methods based on traditional machine learning. The first one covering all types of MoRFs is the MoRFpred [117], released in 2012. Since then, all MoRF predictors have targeted all types of MoRFs without specific distinction. MoRFpred collects a training data set in which the disordered regions are dependent on the disorder prediction; later, some predictors also used this data set for training [64]. Considering the sequence similarity between MoRFs, MoRFpred combines the annotation generated by sequence alignment with the prediction generated by SVM. The input features of SVM are PSSM, the physicochemical properties of amino acids, disorder prediction, predicted flexibility, and solvent accessibility prediction. FMoRFpred [101] uses a scheme similar to MoRFpred; they both choose SVM as a classifier and have similar feature sets. MFSPSSMpred [121] and DISOPRED3 [87] predict MoRFs based on SVM and use sequence-derived features and evolution profiles as input [56]. MoRF_CHiBi_ [122] trained two SVMs, one based on the similarity between MoRFs and the other based on the comparison of amino acid composition between MoRF and its flanking regions. MoRF_CHiBi_ uses the Bayes rule to connect the tendency scores generated by two SVMs to obtain the final prediction, which does not depend on the evolutionary information and the output of other predictors but has good predictive performance [119]. MoRFpred-plus [124] uses two SVM-based propensity scores for prediction. One calculates the composition and similarity of the assumed MoRF region and flank region, and the other calculates the features around a given residue based on the HMM profile. The final prediction of MoRFpred-plus is generated by the two propensity scores.

At present, there are few methods based on deep learning, which need to be further developed. For example, MoRF_CNN_ [119] trained three CNNs using the three feature sets of protein sequences, one from MoRF_MPM_ and the other two from MoRF_CHiBi_. The results of three CNNs are combined to obtain MoRF_CNN_ prediction. In the MoRF_CNN_ method, the sliding window method is still used to extract a $N_{win}\times N_{fea}$ (here $N_{win},N_{fea}$ refer to the number of windows and features) dimension feature vector of each residue instead of inputting the entire sequence into the model to use the global information.

The meta predictors consider the complementarities between different predictors and combine single predictors to produce better predictions than component predictors, such as MoRF_CHiBi_*Light*_ [100], MoRF_CHiBi_*Web*_ [100], OPAL [125], and OPAL+ [126]. Specifically, MoRF_CHiBi_*Light*_ [100] uses the Bayes rule to combine MoRF_CHiBi_ scores with the disorder prediction generated by ESpritz [84], which is more accurate than MoRF_CHiBi_. MoRFs are more conservative than other parts of IDRs, so MoRF_CHiBi_*Web*_ [100] combines MoRF_CHiBi_ prediction, disorder prediction from ESpritz and score generated by the PSSM profile through the Bayes rule to generate the final prediction. Compared with MoRF_CHiBi_*Light*_, MoRF_CHiBi_*Web*_ also uses evolutionary information, so it performs better and runs slower than MoRF_CHiBi_*Light*_. OPAL [125] combines the outputs of two predictors with an average method. One is MoRF_CHiBi_, and the other is a predictor using an SVM based on the structural information of the flanks around MoRFs. Considering that MoRFs with different lengths have different characteristics and the existing methods use a single model to predict MoRFs with different lengths, OPAL+ [126] trains four separate models for MoRFs with different lengths. Finally, OPAL+ combines the four predictor scores, MoRFPred-plus scores and MoRF_chibi_ scores to obtain the MoRF tendency score of each residue in the query region.

Meta predictors can produce better predictive performance than component predictors but at the cost of higher computational cost and more running time.

In the MoRF predictors, the traditional machine learning-based method is still the mainstream. Few of them use deep learning technology, and there is no sequence labeling model. For existing predictors, it may be difficult to extract the interdependencies between residues in the whole sequence.

2. Prediction of short linear motifs (SLiMs)

Short linear motifs (SLiMs) are short and conservative functional sites usually found in the disordered region, with a length of 3–10 amino acids [100,124,127], which can promote biological processes such as cell signal transduction, post-translational modification and protein transportation [127]. Structurally, SLiMs often appear in IDRs at protein termini or between domains and can adopt multiple conformations [128]. Unlike MoRFs, SLiMs exist not only in IDRs but also in the globular protein domain [129]. Although SLiMs and MoRFs overlap, the methods for identifying their locations can be different [124]. Compared with MoRFs, SLiMs have a tendency for convergence evolution, and due to their short length, they are more difficult to predict and prone to high false positives [122,124,127].

Some methods rely on the recognition of over-represented, convergently evolved motifs found in different sequences that bind to common protein partners [101,128], such as DILIMOT [130], SliMFinder [131], SliMDisc [132], etc.

The first de novo method to predict SLiMs specifically is SLiMPred [127], which was proposed in 2012. Based on the types of amino acids, predicted secondary structure, structural motif, solvent accessibility, and disorder, bidirectional RNN (BRNN) is built to predict SLiMs. SLiMPred does not depend on PPI experimental data and can predict SLiMs in ordered and disordered protein sequences at the same time. SLiMPred is a sequence labeling model which gives the probability that each residue is a SLiM residue but cannot identify long binding regions and distinguish SLiMs located in IDRs or globular proteins. Unlike the sequence-based approach, PepBindPred [128] takes into account the protein-protein docking scores and trains a BRNN using protein sequences, predicted secondary structures, AutoDock Vina docking scores, and predicted disorder scores as inputs. Since PepBindPred requires structural information, it may limit the scope of its application.

3. Prediction of disordered protein-binding regions

More generally, some predictors are aimed at the general disordered protein-binding regions (DPBRs) in IDRs, which include MoRF regions. There are three predictors of DPBRs: ANCHOR [11,133], DisoRDPbind [134,135], and ANCHOR2 [56]. ANCHOR is a scoring function-based method focusing on the regions undergoing a disorder-to-order transition that relies on the paired energy estimation method in IURed to calculate three different scores. The final prediction of the binding tendency of residues in the putative disordered region is a linear combination of these three scores. DisoRDPbind calculates the feature vectors of each residue based on the sliding window, which are input into the LR model after feature selection. DisoRDPbind combines regression-based prediction with functional annotation found through sequence similarity to generate the final prediction. In addition to the protein-binding sites of IDRs, DisoRDPbind also predicted the disordered DNA-binding and RNA-binding sites for the first time, which was realized through three LR models. ANCHOR2 is a scoring function-based method that uses a simple and explicable formula based on energy estimation to predict the disordered binding region.

4. Prediction of Linear interacting peptides (LIPs)

LIPs are fragments in protein sequences that undergo disorder-to-order transitions when binding with proteins and nucleic acids [118]. Compared with MoRFs, DPBRs and SLiMs, LIPs are a broader category and a superset of them [118]. CLIP [118] is the first method to predict LIPs from protein sequences. CLIP combines three types of inputs to predict: coevolutionary information, predicted disorder and physicochemical properties of amino acids. Specifically, RF and SVM are trained based on coevolutionary information and the physicochemical properties of amino acids, respectively. The outputs of these two models, together with the disorder prediction and the disorder content of each sequence, are input into the LR model as feature sets to generate LIP prediction. Unlike other predictors, CLIP uses coevolutionary information, which has proven to be useful in ablation experiments. Although without special training, CLIP can also predict SLiMs and MoRFs well compared with other top-performing predictors specially designed for these binding regions.

5. Prediction of Disordered lipid-binding residues (DLBRs)

Considering the growth of experimental annotations of Disordered lipid-binding residues (DLBRs) and the importance of lipid–IDR interaction, Katuwawala et al. [112] first proposed a predictor especially for DLBRs, DisoLipPred. DisoLipPred is based on the deep BRNN, mainly including four modules: bypass module, sequence profile module, deep neural network, and rescaling module. The bypass module inputs the disordered residues predicted by SPOT-Disorder into the depth neural network after being processed by the sequence profile module, while the predicted ordered residues bypass the depth neural network. The rescaling module combines the predictions of the depth network and the ordered residues to obtain the final output. The deep neural network contains the network obtained by transfer learning, which is constructed by using the complete training set without relying on partners.

Transfer learning enables DisoLipPred to make full use of interacting IDRs data, not limited to lipid–IDR interaction, so as to improve the learning ability of the model for DLBRs. The bypass module makes the model pay more attention to the difference between disordered residues and disordered binding residues. They are the main innovation and contribute to the performance of the model, especially the bypass module.

6. Prediction of Semi-disordered regions

Zhang et al. [136] defined the prediction of the semi-disordered state as a region with a disorder or order probability of 50%. These semi-disordered regions are semi-collapsed with some secondary structures, which are related to induced folding and protein aggregation [35,136]. Although there is no specific training, experiments show that the prediction of semi-disordered regions generated by SPINE-D, SPOT-Disorder, and SPOT-Disorder2 can be used to identify MoRFs. The test results on the dataset show that the semi-disordered regions predicted by SPOT-Disorder2 are more accurate in identifying MoRFs than some methods designed directly for identifying MoRFs.

7. Prediction of protein-binding residues

Most of the above predictors only focus on the disordered binding sites but cannot correctly predict the binding sites in ordered proteins, that is, disorder-specific. Similarly, ISIS [137], DeepPPISP [138] and other methods use ordered protein data for training but cannot be applied to the prediction of disordered binding sites. For the defect that the above predictors cannot predict the binding sites of two types of proteins at the same time, Zhang et al. [115] proposed a novel fusion predictor called hybridPBRpred, which combines disoRDPbind and SCRIBER to predict the binding sites of ordered and disordered proteins at the same time.

In Table 4, we have summarized the predictors of disordered binding regions. All of the websites of these methods have been last accessed on 29 December 2022. The development process of these predictors for different prediction targets is not consistent. MoRF predictors are developed first and are also the most researched (accounting for more than 50% of all predictors). MoRF predictors are mostly based on machine learning methods, especially SVM-based methods, and few use deep learning technology. SLiM predictors also started early but only have some limited prediction methods. Other types of predictors appeared later than 2015, with only 1–3 prediction methods for each type. Perhaps due to the late start, these predictors use some advanced deep learning technologies, such as transfer learning. Although the predictors shown in Table 1 are designed for specific types, they also have the ability to predict other types of binding sites. For example, DisoLipPred can effectively predict MoRFs and SLiMs.

#### 3.2.2. Predictive Performance

CAID evaluated the prediction of binding sites in IDRs using the binding regions annotated by DisProt [18]. In Table 5, predictor thresholds are optimized on MCC. Similar to disorder prediction, per-protein classification performance is worse than per-residue performance in MCC, and the difference between them is further verified by Wilcoxon signed-rank test (*p*-value < 0.05). For the prediction of disordered binding sites, there are also some targets (such as DP01108) that are difficult to predict, which may need extra attention in the future. As shown in Table 5, the best MCC of per-protein and per-residue classification are 0.062 and 0.199, respectively, so there is a large room for improvement in the performance of the predictors for disordered binding sites. We noticed that DisoRDPbind-DNA, DisoRDPbind-RNA, and DisoRDPbind do not perform well, which may be caused by the mismatch between the target prediction object of the predictors and the actual prediction object.

Among the 11 submitted methods, the CAID evaluation results show that the top five methods with the best performance are ANCHOR-2, DisoRDPbind, MoRF_CHiBi_ (light and web), and OPAL. These five predictors, except ANCHOR-2, which is based on the scoring function-based method, are all based on machine learning, and especially three of them are meta predictors. Among them, ANCHOR-2 and DisoRPbind secure the best performance, which is probably because they are not limited to short binding regions during training and can adapt to broader prediction targets of CAID.

They can differ by three orders of magnitude in execution times, and the running time is inversely proportional to the performance. The ANCHOR-2 with the best performance requires the shortest running time; this is probably because it is a scoring function-based method and does not involve complex models and calculations.

#### 3.2.3. Problems and Perspectives

First, there is room for improvement in the predictive performance of the predictors. The CAID evaluation results show that the disordered binding region is still difficult to predict, and it is necessary to develop updated and better predictors of binding regions.

In terms of data, more precise experimental annotations of binding sites are needed. The test data used in CAID1 come from the DisProt database, in which the binding annotations retrieved from the literature have more ambiguity than the disorder annotations, and the specific location of the binding regions is usually incorrect [18]. In addition, the data used by many MoRF predictors for training is dependent on the predicted disordered regions rather than the disorder identified by experiments [64]. The acquisition of negative samples is also a challenge, the same as the disorder prediction, and higher quality annotations of non-binding residues are required.

There are some limitations in the prediction target. At present, most of the methods are developed for MoRFs. In addition to MoRFs, the disordered binding sites also include many other types. In the protein-binding residues, SLiMs and DPBRs are both included. The existing methods for predicting disordered protein-binding regions mainly focus on the sites where the disorder-to-order transition occurs. There are also some regions where disorder-to-disorder and context-dependent transitions occur. Only a few methods, such as FuzPred, take these two binding modes into account. In addition, IDPs/IDRs can interact with DNA, RNA, lipids, etc., but there are relatively few predictors for such binding sites, which need further development. Moreover, most predictors can only predict one type of binding regions but cannot predict multiple types of binding regions at the same time.

In terms of the training strategy, most methods predict binding sites in all residues without using known disorder annotation/prediction. For the prediction of binding sites of IDRs, the regions that do not belong to IDRs can be excluded so that only the disordered residues (annotation or prediction) can be used for training and predicting; thus disordered binding sites and disordered residues can be better distinguished. DisoLipPred uses the bypass module for the first time to achieve this purpose.

## 4. Prediction of IDPs/IDRs Functions

DisProt has annotated the functions of disordered regions, most of which are related to binding functions. In addition to the binding function, some predictors have been developed for the other functions of a disorder.

### 4.1. Methods

#### 4.1.1. Prediction of Disordered Flexible Linkers (DFLs)

Disordered flexible linkers (DFLs) are IDRs that serve as flexible linkers/spacers in multi-domain proteins or between structured constituents in domains [139]. DFLs are important for many cellular processes, including amyloid fibril formation and the movement between catalytic sites. DFLpred [139] is the first method to predict DFLs. DFLpred uses the sliding window to calculate the features based on the amino acid type, secondary structure prediction, disorder prediction and sequence complexity of each residue and inputs the selected features into the LR model to obtain the propensity score.

#### 4.1.2. Prediction of Disordered Moonlighting Regions (DMRs)

The high plasticity of IDRs makes it possible for them to combine with a variety of ligands and perform different functions. Disordered moonlighting regions (DMRs) are IDRs that carry out multiple functions [140]. Different from the predictors focusing on specific functions, DMRPred [140] is the first method to predict DMRs. DMRPred generates the features of each residue by calculating the information, including sequence conservation, predicted relative solvent accessibility and disorder, etc., of the target residue and its neighbors, which are input into the RF model to generate predictions.

#### 4.1.3. Prediction of Multi-Functions of Disorder

Most of the current predictors focus on predicting one function of IDRs, and only a few predictors can simultaneously predict multiple functions of IDRs. For example, DisoRDPbind is used to predict protein-binding, DNA-binding, and RNA-binding at the same time. FlDPnn can simultaneously predict four functions: protein-binding, DNA-binding, RDN-binding, and linker.

### 4.2. Problems and Perspectives

Most of the current methods only focus on one kind of function, and the predictors that can simultaneously predict multiple functions need to be further developed. According to the DisProt annotation, the molecular functions of IDRs can be divided into six categories: entropic chains, display sites, chaperons, effectors, assemblers, and scavengers [114]. Therefore, the predictors can be extended to other molecular functions of IDRs.

## 5. Features Used by the Predictors

The research shows that the improved performance of the predictors mainly depends on the input features and profile. Because of the importance of features and the commonality of features used by different predictors, we discussed these features in a separate section. Feature extraction includes information used and feature encoding methods.

The information used in features can be mainly divided into four categories. (i) Sequence information, which is obtained from a single-input sequence. This includes amino acid composition (AAC), reducing amino acid composition (RAAC), physicochemical properties of amino acids (aromaticity, net charge, flexibility, hydrophilicity, coordination number, etc.), correlated contact potential, and the propensity of being at the endpoints of a secondary structure segment, etc. This kind of information is obtained from a single sequence, and the selection of features applies some fields of knowledge, such as low complexity and low content of hydrophobic amino acid residues of the IDR sequence. Such features do not require additional tools for calculation and are widely used in various predictors. (ii) Evolutionary information includes the PSSM and HMM profiles generated by MSA and the whole MSA as text input (such as rawMSA) and BLOSUM62. The use of evolutionary information can improve the predictive performance when the number of homologues is large, but MSA usually requires more search time. (iii) Structural information, mainly generated by other tools, such as predicted secondary structure, torsional angle fluctuation and solvent accessibility, early folding, etc. Because the definition of disorder is related to structure, such information is also widely used. (iv) Prediction results generated by the predictors related to disorder. For example, disorder function predictions and binding region predictions usually use the results of disorder predictors as input. The disorder predictors also use the results of the function predictors as input information, such as flDPnn.

Classification models and sequence labeling models correspond to different feature encoding methods. Classification models usually encode the relevant information of the target residue into a fixed-length vector. The encoding mode can be divided into residue level, window level, and protein level. Residue-level coding method only focuses on the information of the target residue or a few residues around. The window-level coding method uses a sliding window to cover the subsequence centered on the target residue so as to characterize the information of this subsequence (such as taking the average feature values of the subsequence, etc.). This is because the function and structure of the target residue are affected by its neighboring residues. In practical applications, multiple features can be encoded to be used simultaneously according to different window sizes. In addition, another method (svmPRAT) uses a flexible window-based encoding scheme, which weights the input information according to the distance between the adjacent residue and the central residue. Encoding based on the protein level is relatively less used, which includes calculating the average value of features on the whole sequence (such as the disorder content) and the length of the sequence. In the ablation experiment of flDnn, protein-level encoding proved to be very useful.

Sequence labeling models represent the whole sequence numerically rather than focusing on local sequences. The numerical representation method can be divided into using pre-calculated features and embedding methods, which use an embedding layer so that the network automatically extracts features. Using pre-calculated features is to encode each residue on the sequence based on the types of amino acids, the predicted secondary structure, evolutionary profiles, and other information so as to form a feature matrix, and each dimension represents the feature vector of a residue. The embedding method mainly comes from the technology in Natural Language Processing (NLP), such as the training embedding layer, Word2Vec, and GloVe, and is also widely used in the field of bioinformatics. The word embedding method automatically extracts the features of the sequence through the neural network instead of manual empirical feature extraction and selection. For example, rawMSA takes the whole MSA as the text input of the neural network and uses the depth network to perform automatic feature extraction.

From the above summary, we can see that there are many available feature information and encoding methods, but it is not always better to use more features, because too many would cause information redundancy and noise. It is necessary to combine domain knowledge and experiments to carry out feature engineering so as to effectively improve the performance of the model. The use of evolutionary information can improve the predictive performance in some cases, and the methods based on a single sequence are suitable for large-scale screening and also conducive to the study of orphan proteins. The use of evolutionary information has its advantages and disadvantages, but only a few methods give the option of whether to use this information. At present, the existing methods, even those based on deep learning, mostly rely on some features selected by experience and pre-determined profiles to train the prediction model, which may lead to a curse of dimension and difficulty in capturing potential information.

## 6. Future Prospects

In the above, we have discussed the problems and perspectives for different prediction tasks, and we further summarized them in this section.

(i) In terms of datasets, these prediction tasks require more comprehensive and more accurate datasets. At present, most of the predictors that perform better rely on machine learning algorithms, and they are data-driven, especially the deep learning-based methods. More data will help to further improve the predictive performance of the predictors. The datasets also have some limitations and are not comprehensive enough. For example, in disorder prediction, there is a lack of completely structured proteins, and the prediction quality is sensitive to them. The datasets also have accuracy problems. For example, some negative samples are originally from incomplete annotations and are not accurate enough. The annotations of binding regions are ambiguous, and their specific position is inaccurate.

(ii) For different tasks, the predictors need to adapt to broader and comprehensive prediction targets. For example, for IDP/IDR predictors, most of the current algorithms only consider disorder and order. There are differences in the conformation of IDPs/IDRs; however, few predictors focus on more detailed classification. The IDP/IDR predictors perform poorly on some proteins, such as fully disordered proteins and proteins with high disorder content and need to further adapt these proteins and improve the predictive performance. For the prediction of disordered binding regions, most of the predictors are developed only for a subset of binding sites—MoRFs—and only consider the sites where the disorder-to-order transition occurs, while there are few methods for other types of binding sites. For the prediction of the functions of disorder, most predictors only aim at one function of IDPs/IDRs, rather than predict multiple functions at the same time and only involve partial functions of IDPs/IDRs.

(iii) Quality assessment (QA) methods need further development. QA tools are developed to predict which prediction of residue is reliable. However, at present, such QA tools are few and only for partial disorder predictors, not for other types of predictors.

(iv) Models and training methods. In recent years, deep learning has flourished. Some updated deep learning methods such as transformer, attention mechanism, and pre-training model can be considered in the network architecture of predictors. These methods have proven to be effective in the field of biological information [141,142,143], and some are suitable for processing sequential data. In addition, except for its outstanding performance in predicting the three-dimensional structure of proteins, AlphaFold has also been effectively used for PPI prediction [144,145]. AlphaFold has proven to have the ability to predict disordered regions [146]. It is worth considering combining AlphaFold to predict disordered regions, the interaction of IDPs/IDRs, and the molecular functions of IDPs/IDRs.

(v) Use multi-task learning to solve different prediction tasks. Most of the models mentioned above focus on single-task learning, but these tasks have some similarities, such as using similar features and models, and are aimed at disordered regions. Therefore, in view of the similarity of these tasks, multi-task learning [147] in machine learning can be considered to improve learning efficiency and generalization.

**Figure 1:**
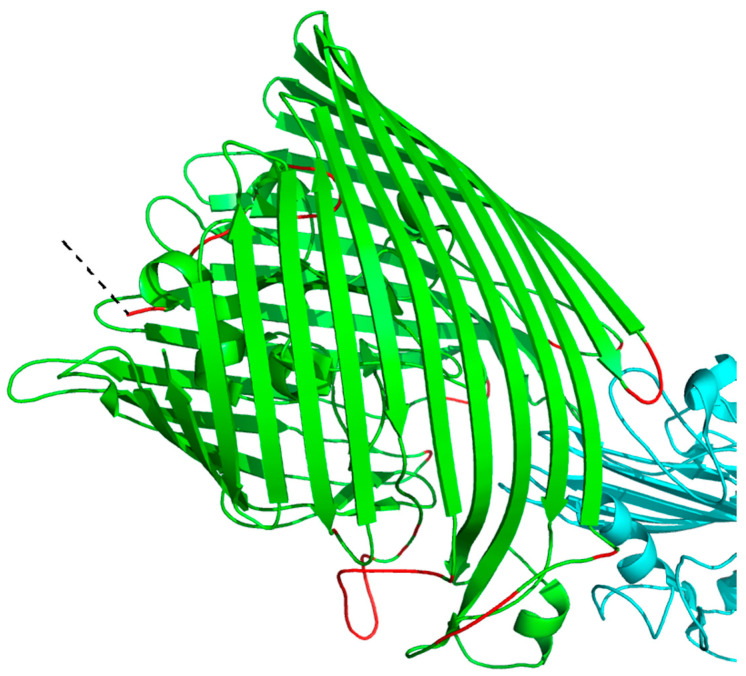
A schematic view of the target H1129 in CASP15 (PDBID:8A8C) with potentially disordered regions. The figure shows the three-dimensional structure of this protein. The potentially disordered regions are marked with red or dotted lines in the figure, where red regions represent the regions of the loop and are predicted to be disordered, and the dotted line represents the region with missing coordinates in the PDB structure and is predicted to be disordered.

As we all know, AlphaFold has made a breakthrough in predicting the three-dimensional structure of proteins. However, for some orphan proteins, the prediction of antibody-antigen interaction sites still cannot replace the experimental results, which forces us to seek other methods to improve the prediction accuracy. In CASP15, there are many targets containing potentially disordered regions, such as target H1129 (as shown in Figure 1). We have combined the study of disorder with the prediction of the three-dimensional structure of protein complexes through different methods. For the sequence to be predicted, we first predicted its disordered regions and then checked whether the secondary structure in the predicted three-dimensional structure conflicts with the predicted disorder, that is, whether the predicted disordered region is a loop region in the predicted structure. If there are more conflicts, it indicates that the structure may not be well predicted. Another method is: in the evaluation of model quality, less attention was paid to the quality of prediction structure in the disordered region, so a more reasonable prediction structure was selected. This also improves the limitations of AlphaFold’s modeling of the loop domain and the inaccurate prediction at the beginning and the end of the sequence. Introducing the research of disordered proteins into the prediction of the three-dimensional structure of proteins is more worthy of expectation in the future.

## 7. Conclusions

The computational methods related to intrinsic disorder have been developed vigorously, especially the prediction of IDRs. Since the first predictor was proposed in 1979, dozens of prediction methods have been developed, from the relatively simple scoring function-based methods to the more complex machine learning-based methods and meta-predictors. The development of these predictors has reached a relatively mature state, but there are still some problems to be solved, which have been discussed above.

The prediction of binding modes of IDPs/IDRs pays attention to disorder-to-disorder, disorder-to-order, and context-dependent transitions. There are few studies that consider all of the above three transitions, while many studies only focus on the type of disorder-to-order.

IDPs/IDRs can interact extensively with proteins, DNA, RNA, lipids, and small molecules. Different methods have been developed to predict the binding sites of IDRs according to different types of binding sites. In addition to considering partner types, protein-binding residues are divided into MoRFs, SLiMs, and general PBDRs. Although many predictors of disordered binding sites have been developed, most of them only focus on MoRF prediction. The predictors of binding sites need to be further improved in terms of the predictive performance and the universality of prediction sites.

There are relatively few methods to predict the molecular functions of IDRs. In addition to binding functions, existing predictors also predict the disordered flexible linkers and disordered moonlighting regions. Most predictors are specially designed for one function and seldom consider coupling disorder prediction and function prediction to ensure the consistency of the results.

## Figures and Tables

**Table 1:** Disorder residues in the datasets.

Dataset	[9]	PDBS25	Seven-Body Proteins	Nine-Body Proteins
No. of proteins	37,395		19	15
No. of chains		1223	133	135
No. of residues	16,492,809	239,527	82,152	28,467
No. of proteins with disorder	19,101		14	7
No. of chains with disorder		696	93	63
Proteins/Chains with disorder (%)	51.08	56.91	69.92	46.67
No. of SDRs	28,829	1100	74.03	46.67
No. of LDRs	3553	68		
No. of IDRs	32,382	1168		
SDRs in IDRs (%)	89.03	94.18		
No. of disordered residues		12,138	4910	1485
Disordered residues (%)		5.07	5.98	5.22

In the dataset of Monzon et al., SDRs are IDRs with length 5+ and <30, and LDRs are IDRs with length ≥30. In PDBS25, SDRs are IDRs with length ≤ 30, and LDRs are IDRs with length > 30. PDBS25 (PDB-Select-25) is a PDB subset selected from the PDB database with homology < 25% and covering the PDB as completely as possible.

**Table 2:** The summary of IDP/IDR predictors.

Predictor	Publication Year	Web Sites	Category	Type of IDRs	Classifier	Features	Whether to Use Evolutionary Information
IUPred2A	2018	http://iupred2a.elte.hu accessed on 29 December 2022	SF	G			No
SPOT-Disorder-Single	2018	http://sparks-lab.org/jack/server/SPOT-Disorder-Single accessed on 29 December 2022	ML	G	residual CNNs, LSTM	Binary one-hot matrix	No
SPOT-Disorder2	2019	https://sparks-lab.org/server/spot-disorder2/ accessed on 29 December 2022	ML	G	LSTM IncReSeNet	Evolutionary information including PSSM, HMM profile and predicted structural properties from SPOT-1D.	Yes
RawMSA	2019	https://bitbucket.org/clami66/rawmsa 29 December 2022	ML	G	LSTM, BRNN, CNN	MSA	Yes
fIDPnn	2021	http://biomine.cs.vcu.edu/servers/flDPnn/ accessed on 29 December 2022	ML	G	DNN	Secondary structure predictions, disorder predictions, disorder predictions, PSSM, conservation scores, prediction of disordered function generated by DisoRDPbind, DFLpred and fMoRFpred.	No
flDPlr	2021		ML		LR	Same as flDPnn	No
IUPred3	2021	https://iupred3.elte.hu accessed on 29 December 2022	SF	Can be divided into IUpred long and IUPred short			No
DisoMine	2022	https://bio2byte.be/disomine/ accessed on 29 December 2022	ML	More suitable for LDRs	GRU	Secondary structure predictions, backbone and side-chain dynamics predictions and early folding propensity predictions	No

In the ‘Category’ column, ‘SF’ represents the scoring-based method, ‘ML’ represents the machine learning-based method, ‘Meta’ represents the meta predictors. In the ‘Type of IDRs’ column, ‘G’ represents that the predictors can predict the general IDRs.

**Table 3:** Predictive performance of disorder predictors in CAID1.

Predictor	DisProt (Per-Protein)			DisProt (Per-Residue)			DisProt-PDB (Per-Protein)			DisProt-PDB (Per-Residue)			Fully IDPs		
	BAC	MCC	F1-S	BAC	MCC	F1-S	BAC	MCC	F1-S	BAC	MCC	F1-S	BAC	MCC	F1-S
SPOT-Disorder2	**0.712**	**0.308**	**0.486**	**0.725**	0.349	0.469	0.753	**0.356**	0.643	0.836	**0.706**	0.784	0.711	0.409	0.452
SPOT-Disorder1	0.706	0.295	0.475	0.723	0.33	0.434	**0.761**	0.33	**0.649**	**0.846**	0.696	**0.788**	0.72	0.416	0.458
RawMSA	0.692	0.29	0.449	0.714	0.328	0.445	0.736	0.299	0.612	0.815	0.635	0.745	0.773	0.546	0.578
AUCpreD	0.704	0.283	0.466	0.712	0.318	0.433	0.738	0.334	0.623	0.816	0.662	0.756	0.678	0.431	0.453
DISOPRED-3.1	0.674	0.267	0.427	0.674	0.258	0.393	0.703	0.306	0.581	0.796	0.613	0.724	0.563	0.246	0.214
Predisorder	0.671	0.263	0.429	0.691	0.301	0.435	0.697	0.303	0.579	0.788	0.619	0.717	0.701	0.479	0.5
IUPred2A-short	0.674	0.256	0.424	0.688	0.29	0.42	0.693	0.27	0.567	0.773	0.574	0.691	0.609	0.413	0.351
IUPred-short	0.675	0.256	0.424	0.688	0.288	0.418	0.7	0.27	0.574	0.775	0.571	0.693	-	-	-
AUCpreD-np	0.681	0.254	0.441	0.699	0.301	0.424	0.722	0.297	0.601	0.797	0.615	0.725	0.602	0.293	0.303
SPOT-Disorder-Single	0.676	0.251	0.44	0.71	0.315	0.432	0.73	0.29	0.608	0.817	0.646	0.751	0.661	0.452	0.448
MobiDB-lite	0.668	0.247	0.423	0.688	0.289	0.42	0.673	0.274	0.55	0.764	0.583	0.683	0.621	0.437	0.379
fIDPnn	0.668	0.247	0.44	0.72	**0.37**	**0.483**	0.713	0.252	0.591	0.782	0.576	0.701	**0.776**	**0.569**	**0.598**
IsUnstruct	0.667	0.244	0.425	0.689	0.288	0.418	0.691	0.272	0.574	0.779	0.585	0.7	0.667	0.411	0.432
IUPred-long	0.654	0.243	0.395	0.686	0.287	0.418	0.679	0.244	0.54	0.783	0.588	0.704	-	-	-
ESpritz-X	0.669	0.241	0.427	0.689	0.288	0.418	0.712	0.272	0.586	0.778	0.566	0.695	0.595	0.321	0.3
VSL2B	0.663	0.24	0.421	0.684	0.277	0.408	0.674	0.264	0.549	0.774	0.581	0.695	0.736	0.468	0.505
IUPred2A-long	0.654	0.24	0.396	0.685	0.285	0.416	0.669	0.242	0.529	0.776	0.584	0.697	0.639	0.42	0.406
JRONN	0.657	0.238	0.404	0.672	0.263	0.401	0.657	0.258	0.528	0.751	0.546	0.661	0.628	0.397	0.381
ESpritz-N	0.647	0.236	0.389	0.664	0.259	0.4	0.661	0.268	0.524	0.751	0.554	0.662	0.63	0.426	0.393
fIDPlr	0.647	0.22	0.409	0.693	0.33	0.452	0.689	0.23	0.562	0.761	0.537	0.671	0.771	0.468	0.505
DynaMine	0.654	0.22	0.4	0.66	0.24	0.384	0.657	0.245	0.527	0.739	0.505	0.641	0.5	0	0
DisoMine	0.643	0.206	0.42	0.698	0.299	0.424	0.693	0.205	0.558	0.78	0.55	0.693	0.77	0.421	0.455
PyHCA	0.64	0.198	0.39	0.66	0.241	0.385	0.642	0.226	0.5	0.731	0.494	0.629	0.629	0.411	0.387
S2D-1	0.61	0.19	0.35	0.633	0.203	0.361	0.603	0.218	0.456	0.724	0.494	0.617	-	-	-
DisEMBL-465	0.608	0.18	0.357	0.627	0.214	0.363	0.61	0.209	0.485	0.694	0.426	0.57	0.522	0.204	0.085
S2D-2	0.618	0.173	0.365	0.624	0.183	0.347	0.649	0.19	0.511	0.703	0.386	0.591	0.643	0.288	0.337
FoldUnfold	0.62	0.169	0.386	0.642	0.211	0.365	0.665	0.176	0.553	0.736	0.462	0.636	0.724	0.256	0.281
ESpritz-D	0.632	0.167	0.4	0.703	0.307	0.428	0.67	0.152	0.534	0.778	0.544	0.69	0.706	0.342	0.389
GlobPlot	0.57	0.136	0.3	0.587	0.143	0.312	0.549	0.16	0.394	0.641	0.328	0.48	0.5	0	0
DisEMBL-HL	0.563	0.135	0.269	0.577	0.172	0.286	0.661	0.132	0.531	0.641	0.262	0.535	0.544	0.288	0.163
DisPredict-2	0.557	0.06	0.31	0.599	0.152	0.326	0.58	0.061	0.435	0.625	0.24	0.491	0.632	0.33	0.356
DFLpred	0.473	0.02	0.033	0.503	0.022	0.025	0.368	0.024	0.046	0.504	0.043	0.027	0.5	0	0

The maximum value of each metric in each dataset is marked in bold. In the first four datasets, predictor thresholds are optimized on MCC.

**Table 4:** Summary of the prediction methods of disordered binding sites.

Target of Predictions	Predictor	Publication Year	Web Site	Category	Classifier or Combination Mode	Features
MoRF regions	α-MoRFpred	2005	NA	ML	Neural network	Disorder predictions, secondary structure predictions, and amino acid index
	Retro-MoRFs	2010	NA	SF		Sequence alignment and disorder predictions
	MoRFpred	2012	http://biomine.cs.vcu.edu/servers/MoRFpred/ accessed on 29 December 2022	ML	SVM	Sequence alignment, PSSM, physicochemical, properties of amino acids, disorder predictions, predicted flexibility, and solvent accessibility prediction
	MFSPSSMpred	2013	The website does not work as of January 2019	ML	SVM	PSSM
	MoRFCHiBi	2015	https://gsponerlab.msl.ubc.ca/software/morf_chibi/ accessed on 29 December 2022	ML	SVM	Similarity between MoRF sequences and amino acid composition
	DISOPRED3	2015	http://bioinf.cs.ucl.ac.uk/disopred accessed on 29 December 2022	ML	SVM	Blosum62 matrix and PSSM
	fMoRFpred	2016	http://biomine.cs.vcu.edu/servers/fMoRFpred /accessed on 29 December 2022	ML	SVM	Putative annotation of intrinsic disorder and secondary structure, estimated B-factor, structural stability, and unfolding energy.
	MoRFCHiBiLight	2016	https://gsponerlab.msl.ubc.ca/software/morf_chibi/ accessed on 29 December 2022	Meta	Bayesian rules	MoRFCHiBi and Espritz
	MoRFCHiBiWeb	2016	https://gsponerlab.msl.ubc.ca/software/morf_chibi/ accessed on 29 December 2022	Meta	Bayesian rules	MoRFCHiBi, Espritz, and the score generated by the PSSM profile
	Predict-MoRFs	2016	https://github.com/roneshsharma/Predict-MoRFs accessed on 29 December 2022	ML	SVM	HMM
	MoRFPred-plus	2018	https://github.com/roneshsharma/MoRFpred-plus/wiki/MoRFpred-plus accessed on 29 December 2022	ML	SVM	Physicochemical properties and HMM profile
	OPAL	2018	http://www.alok-ai-lab.com/tools/opal/ accessed on 29 December 2022	Meta	An average method	MoRFCHiBi and an SVM model based on the structural information
	OPAL+	2018	http://www.alok-ai-lab.com/tools/opal_plus/ accessed on 29 December 2022	Meta	The common averaging principle	Four SVMs trained for MoRFs with different lengths, MoRFPred-plus and MoRFchibi
	MoRFCNN	2021	NA	ML	CNNs	Three feature sets, one from MoRFMPM and two feature sets from MoRFCHiBi.
SLiMs	SLiMPred	2012	http://bioware.ucd.ie/~compass/biowareweb/Server_pages/slimpred.php accessed on 29 December 2022	ML	Bidirectional RNN	Predicted secondary structure, structural motif, solvent accessibility and disorder
	PepBindPred	2013	http://bioware.ucd.ie/~compass/biowareweb/Server_pages/pepbindpred.php accessed on 29 December 2022	ML	Bidirectional RNN	Protein sequences, predicted secondary structures, AutoDock Vina docking scores, and predicted disorder scores
DPBRs	ANCHOR	2009	http://anchor.enzim.hu accessed on 29 December 2022	SF		Paired energy estimation
	DisoRDPbind	2015	http://biomine.cs.vcu.edu/servers/DisoRDPbind/ accessed on 29 December 2022	ML	Logistic regression	Functional annotation, amino acid composition, sequence complexity, prediction of disorder, prediction of secondary structure, and physicochemical properties of amino acids
	ANCHOR2	2018	http://iupred2a.elte.hu accessed on 29 December 2022	SF		Interactions and disordered environment
LIPs	CLIP	2022	http://biomine.cs.vcu.edu/servers/CLIP/ accessed on 29 December 2022	ML	RF and SVM	Coevolutionary information, predicted disorder, disorder content and physicochemical properties of amino acids
DLBRs	DisoLipPred	2022	http://biomine.cs.vcu.edu/servers/DisoLipPred/ accessed on 29 December 2022	ML	Bidirectional RNN	Predicted secondary structure, solvent accessibility, disorder, disorder function, physicochemical properties of amino acids
Semi-disorder	SPINE-D	2013	http://sparks-lab.org/SPINE-D/ accessed on 29 December 2022	ML	Neural network	Physical parameters, PSSM, predicted secondary structure, predicted solvent accessibility, predicted torsional angle fluctuation, amino acid composition, local composition complexity and predicted secondary structure content.
	SPOT-Disorder	2017	http://sparks-lab.org/server/SPOT-disorder/ accessed on 29 December 2022	ML	LSTM	Evolutionary information, predicted structural properties and physicochemical properties
	SPOT-Disorder2	2019	https://sparks-lab.org/server/spot-disorder2/ accessed on 29 December 2022	ML	IncReSeNet, LSTM and Full Connect	Similar to SPOT-Disorder
protein-binding residues	hybridPBRpred	2020	http://biomine.cs.vcu.edu/servers/hybridPBRpred/ accessed on 29 December 2022	Meta	A hybrid approach	SCRIBER and disoRDPbind

In the ‘Category’ column, ‘SF’ represents the scoring-based method, ‘ML’ represents the machine learning-based method, ‘Meta’ represents the meta predictors.

**Table 5:** The performance of predictors of binding regions in CAID.

Predictor	Per-Protein Classification			Per-Residue Classification		
	BAC	MCC	F1-S	BAC	MCC	F1-S
DisoRDPbind-protein	0.652	**0.062**	0.137	**0.697**	0.198	0.214
ANCHOR-2	0.677	0.055	0.13	0.694	**0.199**	**0.22**
MoRFchibi-light	0.629	0.041	0.124	0.636	0.161	0.212
MoRFchibi-web	0.588	0.039	0.131	0.631	0.143	0.194
OPAL	0.482	0.039	**0.141**	0.652	0.151	0.186
DISOPRED-3.1-binding	0.725	0.036	0.095	0.569	0.099	0.169
ANCHOR	0.571	0.026	0.127	0.651	0.148	0.178
fMoRFpred	0.79	0.014	0.031	0.515	0.054	0.072
DisoRDPbind-DNA	**0.804**	0.004	0.005	0.502	0.052	0.008
DisoRDPbind-RNA	0.799	0.002	0.007	0.501	0.014	0.01
DisoRDPbind	0.194	0	0.131	0.5	0	0.119

The maximum value of each metric in each dataset is marked in bold.

## Data Availability

Data availability is not applicable to this article as no new data was created or analyzed in this study.
